# A Retrospective Observational Study on Disease Characteristics and Treatment Patterns of Giant Cell Tumor of the Bone in China

**DOI:** 10.1155/2023/5468291

**Published:** 2023-04-28

**Authors:** Hairong Xu, Yuan Li, Tao Wang, Weifeng Liu, Ke Ma, Yongkun Yang, Zhen Huang, Chuang Li, Xiaohui Niu

**Affiliations:** ^1^Department of Orthopaedic Oncology Surgery, Beijing Ji Shui Tan Hospital, Beijing, China; ^2^Amgen China, Shanghai, China

## Abstract

**Aims:**

Giant cell tumor of the bone (GCTB) is associated with considerable morbidity. As GCTB epidemiological data for China are limited, this study is aimed at describing the disease characteristics of GCTB in China and establishing the historical context for its treatment before recent advances in treatment options.

**Methods:**

The disease characteristics, treatment patterns, and local GCTB recurrence rate after primary surgery for GCTB were evaluated in this single-center, retrospective, noninterventional, observational study of patients treated for GCTB at Ji Shui Tan Hospital, Beijing, from 2009 to 2016 based on medical chart review. Patients with unmet need were defined as those whose surgical treatment was difficult or who had to undergo high-morbidity surgery.

**Results:**

Among the 668 patients with a primary GCTB diagnosis, 578 (86.5%) of target lesions were in the extremities, and 89 (13.3%) were in the pelvic or axial bone. Of these, 173 (25.9%) were characterized as having an unmet need. Almost all GCTB patients received surgical treatment at both primary diagnosis (666/668 (99.7%)) and last disease recurrence (196/200 (98.0%)). Additionally, about one-third of patients received nonsurgical treatment at primary diagnosis (205/668 (30.7%)) and disease recurrence (67/200 (33.5%)), with neoadjuvant therapy being the most common treatment. The rate of high-morbidity surgery increased for recurrent disease (65/200 (32.5%)) compared with primary diagnosis (111/668 (16.6%)). The 2-year cumulative incidence of postoperative disease recurrence was 29.2%, in line with rates observed in prior studies.

**Conclusion:**

As many patients with primary and recurrent disease received high-morbidity surgery, more effective treatments are needed.

## 1. Introduction

Giant cell tumor of the bone (GCTB) is a rare, nonmalignant, but locally aggressive neoplasm of the bone [1]. Approximately 4% to 5% of all primary bone tumors and 20% of primary nonmalignant bone tumors worldwide are due to GCTB [2, 3]. In China, however, GCTB accounts for up to 20% of all primary bone tumors [4]. Young adults are most affected by the disease, with most patients between 20 and 40 years of age [5].

GCTB is characterized by a gradual destruction of the bone, leading to deformity of the joints and disability [6]. The goals of treatment in GCTB are to eradicate the tumor, preserve limb function, and prevent local recurrence and distant metastases [5]. For discrete lesions that have not extended into the soft tissue, local curettage is the standard treatment [1, 7, 8]. However, when more aggressive surgical procedures (e.g., joint resection, joint replacement, amputation, and hemipelvectomy) are performed to minimize the risk of recurrence, they are often associated with high levels of morbidity and disability [9].

Surgery is the mainstay of treatment for GCTB [10]. While surgical management is an effective strategy for most patients, there are two segments of the GCTB patient population whose medical needs remain unmet: patients whose tumor location means they are not suitable surgical candidates (e.g., patients with axial disease) and patients whose disease necessitates surgery that, if undertaken, would lead to severe morbidity (e.g., joint resection, joint replacement, or amputation).

While GCTB data for some countries are published [11, 12], few population-based cancer registries record the incidence of nonmalignant bone tumors; currently, there is no such registry in China that captures these data at a national level. Previous studies investigating the epidemiology of GCTB in China have been limited to patients with disease of the extremities [13–15] or patients from a specific geographic region [14]. The last comprehensive epidemiological survey of GCTB in Chinese patients was published in 1982 at Ji Shui Tan (JST) Hospital [4].

JST Hospital is the leading bone tumor treatment center in China, treating not only those who reside in Beijing but also patients residing in other cities and provinces, such as Hebei province, Henan province, Inner Mongolia Autonomous Region, Shandong province, and Shanxi province. Thus, the hospital treats a geographically diverse patient group that represents a broad cross-section of the Chinese population. With no national Chinese GCTB disease registry in place, the JST Hospital provides a valuable source of GCTB patient data for analysis, which is generalizable to the broader Chinese clinical setting.

To establish the historical context of practice patterns, this retrospective, noninterventional, observational study is aimed at describing the disease characteristics and treatment patterns for GCTB in China in clinical practice before the approval of denosumab in 2019 [16, 17].

## 2. Materials and Methods

### 2.1. Study Design and Participants

This study was a single-center, retrospective, noninterventional, observational study based on medical chart review of JST Hospital records from 2009 to 2016. This study was conducted in accordance with the Declaration of Helsinki and Good Clinical Practice principles. The protocol was approved by a local institution review board and ethics committee (approval number: JST-EC-20170505), and patients provided written informed consent for participation in the study; written consent could be waived in cases of death, unable to contact patient, or oral consent.

Individual patient records (hard copy and/or electronic) were collected using a defined electronic case report form and retrospectively analyzed by study investigators and medical specialist staff from August 2017 to March 2018.

Chinese patients who were diagnosed with and treated for GCTB at JST Hospital from 2009 to 2016 were eligible for study enrolment. Documentations of age at diagnosis, sex, date of diagnosis, anatomic site(s) of disease, and date(s) and type(s) of surgical and nonsurgical treatment were required within the included medical records. For patients with recurrent disease, this information was required at both diagnosis of primary disease and at treatment of last disease recurrence. In addition, for the patients included in the treatment outcome evaluation, at least one documented posttreatment assessment was required for each surgical/nonsurgical treatment event performed.

Patients were excluded from the study if they were diagnosed with primary malignant GCTB or if they received drugs not approved by the China Food and Drug Administration for treatment of GCTB before the end of 2016.

### 2.2. Outcome Assessment

For the analysis, the variables collected from the patient medical records included patient demographics, disease characteristics, date of diagnosis, primary or recurrent disease, localized or metastatic disease, pathological diagnosis, and treatment type.

Disease recurrence data was captured only for patients who underwent surgical treatment and was defined by physicians' judgment and supportive imaging data. For patients with no documented recurrence of GCTB by the analysis cutoff date, time to recurrence was censored at their last contact date.

Where documented in the patient record, a Musculoskeletal Tumor Society (MSTS) score [18] was collected for each surgical treatment performed to facilitate measurement of postoperative functional outcomes. Campanacci imaging grade data were not collected.

### 2.3. Data Assessment and Statistical Analysis

For the analysis, patients were organized into several data sets. The full analysis set (FAS) comprised all patients who fulfilled the inclusion and exclusion criteria; the efficacy analysis set (EAS) included only those patients who underwent surgery and who had at least one documented postoperative assessment; the sufficient follow-up subset (SFS) included only those patients in the EAS with >2 years of follow-up after surgery; and the unmet medical need analysis set (UMNAS) included all patients in the FAS with a tumor site or procedure type associated with a poor prognostic outcome (e.g., metastatic disease, pelvic or axial disease, or resectable disease with unacceptable morbidity).

Continuous outcome variables were summarized by the nonmissing sample size, mean, standard deviation, median, first and third quartiles, minimum, and maximum. Categorical outcome variables were summarized by the nonmissing sample size and the proportion in each category. When needed, the 95% confidence interval (CI) using binomial exact method was provided for the proportion. Time-to-event outcomes were summarized with Kaplan-Meier curves, Kaplan-Meier proportions at selected time points, Kaplan-Meier quartiles and 95% CI (if estimable), the number of patients with events, and the number of patients censored. The cumulative incidence of disease recurrence was estimated using the Kaplan-Meier method.

The effects of the following covariates on disease recurrence were evaluated: age group at primary GCTB diagnosis (<18 years, 18–40 years, and >40 years), sex, hospital where the primary diagnosis of GCTB was made (JST vs. non-JST), and place of residence (Beijing vs. other).

To assess whether the follow-up duration influenced the clinical outcomes, the time to recurrence and the 2-year cumulative incidence of recurrence were analyzed for both primary disease and last disease recurrence, based on the EAS and SFS populations.

## 3. Results

### 3.1. Participants

Of the 732 patients with GCTB who received treatment at JST Hospital from 2009 to 2016, 668 patients (91.3%) met all study eligibility criteria and were included in the FAS. Every patient in the FAS had a recorded primary GCTB diagnosis, and 200 patients in the FAS (29.9%) also had a documented GCTB recurrence. Of the total FAS population, 627 (93.9%) patients were included in the EAS, 249 (37.3%) patients were included in the SFS, and 242 (36.2%) patients were included in the UMNAS.

Deviations from the protocol occurred in nine patients (1.4% of the FAS) who were diagnosed with benign GCTB at JST Hospital but, due to patient noncompliance, did not ultimately receive treatment for the condition at JST Hospital. These were deemed to be minor protocol deviations during the medical review, so these patients were retained in the FAS.

In the FAS, the median overall follow-up duration was 29.7 months; the median follow-up duration was 22.2 months and 44.9 months for patients who received their initial GCTB diagnosis at JST Hospital and non-JST hospitals, respectively.

### 3.2. Demographics

Patients were from 30 regions (provinces) (Table 1(a)). The mean age in the FAS was 30 years, with most patients in the 18 to 40 year age group. Among the FAS patient population, 469 (70.2%) patients were first diagnosed with GCTB at JST Hospital, and 199 (29.8%) patients received their primary GCTB diagnosis at non-JST hospitals. Demographics and baseline characteristics of the UMNAS population were similar to those of the FAS population (Table 1(b)).

### 3.3. Primary Outcomes

#### 3.3.1. Disease Characteristics

In the FAS population with a primary GCTB diagnosis, 578 (86.5%) target lesions were in the extremities, and 89 (13.3%) were in the pelvic or axial bone. In the UMNAS population with a primary GCTB diagnosis, 102 (59.0%) target lesions were in the extremities, and 70 (40.5%) were in the pelvic or axial bone. In recurrent disease, most target lesions were in the extremities for both the FAS and UMNAS populations (Table 2).

#### 3.3.2. Treatment Patterns

Almost all patients in the FAS population received surgical treatment for their primary diagnosis (666/668 (99.7%)) and last disease recurrence (196/200 (98.0%)); the most common procedures were curettage and resection. Among the FAS population, high-morbidity surgery such as joint resection, joint replacement, or amputation was performed on 111 (16.6%) patients with a primary GCTB diagnosis and 65 (32.5%) patients with disease recurrence. All patients who underwent high-morbidity surgery in the FAS were in UMNAS (Table 3).

Additionally, approximately one-third of the FAS population received nonsurgical treatment at both primary diagnosis (205/668 (30.7%)) and disease recurrence (67/200 (33.5%)). The most common nonsurgical treatment was neoadjuvant therapy, which was used at primary diagnosis and for recurrent disease, in 131 (19.6%) and 27 (13.5%) patients, respectively (Table 3). At last disease recurrence, 61 (91.0%) patients received diphosphonate treatment, 8 (11.9%) received serial embolization, 2 (3.0%) received radiation, and 1 (1.5%) received chemotherapy.

In the UMNAS population, nonsurgical treatment was used to an even greater extent, with about half of all patients at primary diagnosis (91/173 (52.6%)) and disease recurrence (37/80 (46.3%)) receiving nonsurgical treatment, with neoadjuvant therapy being the most common nonsurgical treatment.

### 3.4. Secondary Outcomes

In the EAS population, the 2-year cumulative incidence of postoperative disease recurrence was 29.2% (95% CI 25.3, 33.5). In patients who received their initial GCTB surgery at JST Hospital, the 2-year cumulative incidence recurrence was 5.4% (95% CI 3.3, 8.5), whereas it was 68.0% (95% CI 61.3, 74.6) among patients who underwent primary surgical treatment at a non-JST hospital (Table 4).

The 2-year cumulative incidence of disease recurrence was higher in patients who underwent curettage (37.0% (95% CI 32.1, 42.3) than in those who underwent a resection (6.4% (95% CI 3.3, 12.2)). No patients in the EAS population underwent amputation surgery.

Covariate analysis based on the Cox proportional hazard regression of the EAS population found that several factors had a significant impact on patients' postoperative recurrence-free survival. Variables that were associated with a significantly reduced risk of postoperative GCTB recurrence included initial GCTB diagnosis completed at JST Hospital (HR 0.06, 95% CI 0.04, 0.10), primary GCTB diagnosis occurring in 2010 and later (HR 0.52, 95% CI 0.39, 0.70), residence in Beijing (HR 0.43 95% CI 0.27, 0.70), high-morbidity surgery (HR 0.08, 95% CI 0.03, 0.23), and bone reconstruction surgery (HR 0.24 95% CI 0.18, 0.32).

Findings of the sensitivity analysis of the SFS population with longer follow-up were consistent with those of the EAS analysis.

### 3.5. Exploratory Outcomes

The average MSTS score was lower in patients whose target lesions were in axial bone than in patients with lesions in the extremities (Table 5).

Among patients with recurrent disease, there was no shift in the anatomic site of local disease between primary GCTB diagnosis and last disease recurrence in the FAS population: at primary diagnosis, the anatomic site was axial bone for 11 (5.5%) patients and extremities for 189 (94.5%) patients. In the FAS population, rates of metastasis increased from 1.8% of at primary diagnosis of GCTB to 8.0% at disease recurrence (Table 2).

There was an obvious shift in GCTB treatment patterns in the FAS population between primary diagnosis and last disease recurrence. Curettage was performed in 457 (68.4%) patients at primary diagnosis, but only in 94 (47.0%) patients at last disease recurrence. Resection was used to treat 207 (31.0%) patients at primary diagnosis, increasing to half of all patients (98/200 (49.0%)) at last disease recurrence, and while only 1 (0.2%) patient underwent amputation at primary diagnosis, this procedure was performed on 4 patients (2.0%) at disease recurrence. The incidence of high-morbidity surgery almost doubled from primary diagnosis (111/668 (16.6%)) to disease recurrence (65/200 (32.5%)). A similar shift in treatment pattern was seen in the UMNAS population, with patients more likely to undergo high-morbidity surgeries at disease recurrence (65/80 (81.3%)) than at primary diagnosis (111/173 (64.2%)).

## 4. Discussion

In this observational study, surgery was found to be the preferred treatment approach for patients in China with GCTB at both primary diagnosis and for recurrence of disease. The high prevalence of surgical treatment in this study is consistent with earlier findings from Chinese [4, 13, 14] and international GCTB studies [8, 19–21].

GCTB recurrence rates from previous studies varied greatly (10% to 65%) and were contingent on GCTB tumor characteristics and the type of surgery performed [19, 22–26]. The postoperative recurrence of GCTB in this study of 29.2% was within the expected range. Of note, when the primary GCTB diagnosis was treated at JST Hospital, recurrence rates were lower than recurrence rates at non-JST hospitals (5.4% vs. 68.0% at 2 years). This difference could be attributed to underlying patient characteristics, quality-of-care discrepancies, or referral patterns from non-JST hospitals to JST Hospital because of disease progression. It would be interesting if future studies could capture the referral rates to determine if the standardized operation procedures and follow-up care provided at JST Hospital did, in fact, lead to better outcomes.

There is a considerable unmet need for patients with GCTB who do not qualify for surgery due to tumor location or who require high-morbidity surgery. This patient group, represented by the UMNAS group in this study, comprised a substantial proportion of the study population: 25.9% (173/668) of the FAS population at primary GCTB diagnosis, increasing to 40.0% (80/200) of the FAS with recurrent disease. For this sizeable patient group, there was no standard alternative treatment at the time of this study, which occurred before the approval of denosumab for GCTB in China. GCTB produces receptor activator of nuclear kappa-B ligand (RANKL) and is dependent upon it for growth [1]. Denosumab, a RANKL inhibitor, is effective in reducing tumor size when tumors cannot be surgically removed or when surgical resection is likely to lead to severe morbidity [8, 9, 27, 28]. This study may serve as an important historical baseline for future studies evaluating the role of denosumab in addressing the unmet needs of patients in China with a tumor site or procedure type associated with poor prognostic outcomes.

### 4.1. Study Limitations

Despite the diverse nature of the patients treated at JST Hospital, this retrospective, single-center study may not be a comprehensive representation of GCTB disease characteristics and treatment patterns across China. In addition, the high recurrence rate recorded for patients diagnosed at non-JST hospitals is likely biased as patients who received their primary diagnosis at non-JST hospitals presented to JST Hospital only on relapse. Given the surgical focus of JST Hospital, patients who were deemed to be more suitable for nonsurgical treatments may have been referred to other treatment centers and lost to follow-up. As the study inclusion criteria required a minimum level of treatment information for patients, a selection bias may have been introduced if patients refused treatment after receiving their primary diagnosis or if disease recurrence went unreported.

## 5. Conclusions

This retrospective analysis gives an overview of GCTB disease characteristics and treatment patterns in China before the approval of denosumab therapy for GCTB. While this is a single-center study, the highly representative nature of the JST Hospital patient population offers an insight into the epidemiology and management of GCTB across China. The prevalent treatment for GCTB in China is surgery, which is effective for many patients; however, there remains a significant proportion of GCTB patients in China with unmet medical needs, comprising 40% of patients with recurring disease. Alternative treatment options, such as denosumab, are needed to facilitate surgery or provide an alternative treatment option for patients who are not candidates for surgery.

## Figures and Tables

**(a):** 

Region	Frequency	Percent
Anhui province	24	3.59
Beijing	115	17.22
Fujian province	11	1.65
Gansu province	4	0.60
Guangdong province	6	0.90
Guangxi Zhuang Autonomous Region	3	0.45
Guizhou province	6	0.90
Hainan province	3	0.45
Hebei province	104	15.57
Henan province	55	8.23
Heilongjiang province	33	4.94
Hubei province	11	1.65
Hunan province	12	1.80
Jilin province	10	1.50
Jiangsu province	11	1.65
Jiangxi province	18	2.69
Liaoning province	34	5.09
Inner Mongolia Autonomous Region	67	10.03
Ningxia Hui Autonomous Region	3	0.45
Shandong province	47	7.04
Shanxi province	51	7.63
Shaanxi province	6	0.90
Shanghai	2	0.30
Sichuan province	4	0.60
Tianjin	7	1.05
Tibet Autonomous Region	1	0.15
Xinjiang Uygur Autonomous Region	5	0.75
Yunnan province	2	0.30
Zhejiang province	11	1.65
Chongqing	2	0.30

**(b):** 

Item	FAS (*N* = 668)		UMNAS (*N* = 242)	
	Primary diagnosis (*n* = 668)	Last disease recurrence (*n* = 200)	Primary diagnosis (*n* = 173)	Last disease recurrence (*n* = 80)
Age, median (years)	30.0	27.0	30.0	28.0
Age group, *n* (%)				
<18	25 (3.7)	8 (4.0)	7 (4.1)	1 (1.3)
18 to 40	495 (74.1)	160 (80.0)	127 (73.4)	64 (80.0)
>40	148 (22.2)	32 (16.0)	39 (22.5)	15 (18.8)
Sex, *n* (%)				
Male	347 (52.0)	108 (54.0)	91 (52.6)	44 (55.0)
Female	321 (48.1)	92 (46.0)	82 (47.4)	36 (45.0)
Place of residence, *n* (%)				
Beijing	115 (17.2)	19 (9.5)	22 (12.7)	4 (5.0)
Others	553 (82.8)	181 (90.5)	151 (87.3)	76 (95.0)
Hospital where GCTB was first diagnosed, *n* (%)				
JST	469 (70.2)	24 (12.0)	152 (87.9)	8 (10.0)
Non-JST	199 (29.8)	176 (88.0)	21 (12.1)	72 (90.0)

FAS = full analysis set; GCTB = giant cell tumor of the bone; JST = Ji Shui Tan; UMNAS = unmet medical needs analysis set.

**Table 2:** Disease characteristics (FAS and UMNAS).

Item	Description	FAS (*N* = 668)		UMNAS (*N* = 242)	
		Primary diagnosis (*n* = 668)	Last disease recurrence (*n* = 200)	Primary diagnosis (*n* = 173)	Last disease recurrence (*n* = 80)
Target lesion anatomic site, *n* (%)	Extremities				
	Upper limbs	116 (17.4)	40 (20.0)	24 (13.9)	12 (15.0)
	Lower limbs	462 (69.2)	149 (74.5)	78 (45.1)	59 (73.8)
	Axial bone				
	Spine	55 (8.2)	7 (3.5)	55 (31.8)	7 (8.8)
	Pelvis	34 (5.1)	4 (2.0)	15 (8.7)	2 (2.5)

Metastasis, *n* (%)	Yes	12 (1.8)	16 (8.0)	12 (6.9)	16 (20.0)
	No	656 (98.2)	184 (92.0)	161 (93.1)	64 (80.0)

Spine or sacrum GCTB or metastatic GCTB, *n* (%)	Yes	66 (9.9)	23 (11.5)	66 (38.2)	23 (28.8)
	No	602 (90.1)	177 (88.5)	107 (61.9)	57 (71.3)

Pathological diagnosis, *n* (%)	BGCT	668 (100.0)	200 (100.0)	173 (100.0)	80 (100.0)

Secondary malignancy diagnosis	MGCT		1 (0.50)		
	Malignant mesenchymal tumor		1 (0.50)		1 (12.5)
	Undifferentiated small cell sarcoma		1 (0.50)		1 (12.5)

BGCT = benign giant cell tumor; FAS = full analysis set; GCTB = giant cell tumor of the bone; MGCT = malignant giant cell tumor; UMNAS = unmet medical needs analysis set.

**Table 3:** Treatment patterns.

Description	FAS (*N* = 668)		UMNAS (*N* = 242)	
	Primary diagnosis (*n* = 668)	Last disease recurrence (*n* = 200)	Primary diagnosis (*n* = 173)	Last disease recurrence (*n* = 80)
Surgical treatment, *n* (%)	666 (99.7)	196 (98.0)	172 (99.4)	77 (96.3)
Curettage, *n* (%)	457 (68.4)	94 (47.0)	36 (20.8)	8 (10.0)
Resection, *n* (%)	207 (31.0)	98 (49.0)	135 (78.0)	65 (81.3)
Amputation, *n* (%)	1 (0.2)	4 (2.0)	1 (0.6)	4 (5.0)
Others, *n* (%)	1 (0.2)	0 (0.0)	0 (0.0)	0 (0.0)
Any high-morbidity surgery, *n* (%)^∗^	111(16.6)	65 (32.5)	111 (64.2)	65 (81.3)
Nonsurgical treatment by objective, *n* (%)	205 (30.7)	67 (33.5)	91 (52.6)	37 (46.3)
Salvage treatment alone, *n* (%)	2 (0.3)	4 (2.0)	1 (0.6)	3 (3.8)
Neoadjuvant therapy, *n* (%)	131 (19.6)	27 (13.5)	48 (27.8)	15 (18.8)
Adjuvant therapy, *n* (%)	30 (4.5)	22 (11.0)	12 (6.9)	13 (16.3)
Neoadjuvant+adjuvant therapy, *n* (%)	42 (6.3)	14 (7.0)	30 (17.3)	6 (7.5)
Nonsurgical treatment+surgical treatment, *n* (%)^†^	0 (0.0)	1 (0.5)	0 (0.0)	0 (0.0)
Nonsurgical treatment by category, *n* (%)	205 (30.7)	67 (33.5)	91 (52.6)	37 (46.3)
Radiotherapy	18 (2.7)	2 (1.0)	18 (10.4)	2 (2.5)
Bisphosphonates	180 (26.9)	61 (30.5)	66 (38.2)	33 (41.3)
Chemotherapy	0 (0.0)	1 (0.5)	0 (0.0)	1 (1.3)
Serial arterial embolization	45 (6.7)	8 (4.0)	43 (24.9)	6 (7.5)

^∗^Includes joint resection/joint replacement, amputation, or other surgical procedure leading to severe morbidity after surgery. ^†^First-line nonsurgical treatment failed, so the patient was subsequently treated with surgery. FAS = full analysis set; UMNAS = unmet medical needs analysis set.

**Table 4:** Postoperative recurrence-free survival for primary GCTB diagnosis (EAS).

Item	*N*	Event, *n* (%)	Event censored, *n* (%)	Median time to recurrence (months)	2-year recurrence rate (%) (95% CI)
Total	627	197 (31.4)	430 (68.6)	75.8	29.2 (25.3, 33.5)
Sex					
Male	322	107 (33.2)	215 (66.8)	72.2	28.7 (23.5, 34.7)
Female	305	90 (29.5)	215 (70.5)	92.4	29.7 (24.2, 36.2)
Age group					
<18	25	9 (36.0)	16 (64.0)	92.4	31.2 (16.0, 55.0)
18 to 40	475	157 (33.1)	318 (66.9)	72.2	29.7 (25.3, 34.7)
>40	127	31 (24.4)	96 (75.6)	130.9	26.9 (18.8, 37.6)
Place of residence					
Beijing	112	18 (16.1)	94 (83.9)	NE	16.4 (10.0, 26.4)
Others	515	179 (34.8)	336 (65.2)	58.5	31.8 (27.5, 36.7)
Hospital where GCTB was first diagnosed					
JST	436	29 (6.7)	407 (93.3)	NE	5.4 (3.3, 8.5)
Non-JST	191	168 (88.0)	23 (12.0)	16.5	68.0 (61.3, 74.6)
Surgery type					
Curettage	442	183 (41.4)	259 (58.6)	45.4	37.0 (32.1, 42.3)
Resection	185	14 (7.6)	171 (92.4)	NE	6.4 (3.3, 12.2)
Amputation	0	0 (0.0)	—	—	—
Others	0	0 (0.0)	—	—	—
Target lesion anatomic site					
Axial bone	78	14 (17.9)	64 (82.1)	NE	17.6 (9.7, 30.7)
Extremities	548	183 (33.4)	365 (66.6)	72.2	30.8 (26.6, 35.5)

CI = confidence interval; EAS = efficacy analysis set; GCTB = giant cell tumor of the bone; JST = Ji Shui Tan; NE = nonestimable.

**Table 5:** Postoperative MSTS scores (FAS).

Surgery type	*N*	Postoperative MSTS score	
		Mean (SD)	Average mean ratio (SD)
Total	75	26.5 (3.08)	0.19 (0.02)
Surgery			
Curettage	45	26.8 (3.23)	0.19 (0.03)
Resection	30	26.0 (2.83)	0.19 (0.02)
Amputation	0	0 (0.0)	0 (0.0)
Others	0	0 (0.0)	0 (0.0)
Target lesion anatomic site			
Axial bone	5	24.8 (3.90)	0.20 (0.03)
Extremities	70	26.6 (3.01)	0.19 (0.02)

MSTS = Musculoskeletal Tumor Society; SD = standard deviation.

## Data Availability

The data that support the findings of this study are available on request from the corresponding author. The data are not publicly available due to privacy or ethical restrictions.
